# Intestinal ischemia due to methamphetamine use: A case report

**DOI:** 10.1016/j.ijscr.2019.03.061

**Published:** 2019-04-07

**Authors:** Kihoon Choi, Andrew Imrie, Rohan Lourie, Trent Cross

**Affiliations:** a Department of General Surgery, Mater Hospital Brisbane, Raymond Terrace, South Brisbane, Queensland, 4101, Australia; b Department of Radiology, Mater Hospital Brisbane, Raymond Terrace, South Brisbane, Queensland, 4101, Australia; c Department of Anatomical Pathology, Mater Hospital Brisbane, Raymond Terrace, South Brisbane, Queensland, 4101, Australia

**Keywords:** Case report, Methamphetamine, Intestinal ischemia, Intestinal gangrene

## Abstract

**Introduction:**

Methamphetamine use is a rare cause of intestinal ischemia but is of clinical significance due to its high morbidity and mortality. Knowledge of methamphetamine-induced intestinal ischemia has been limited to few case reports.

**Case presentation:**

We describe the case of a 48-year-old man who presented with ischemic bowel related to methamphetamine use. With concern for intestinal infarction the patient was taken to the operating room for emergency laparotomy, which found a segmental gangrene of small bowel and colon. The patient subsequently underwent right hemicolectomy and small bowel resection for damage control, followed by second-look laparotomy and anastomosis. The patient recovered well from the surgery and was discharged without complications.

**Conclusion:**

This case report alerts surgeons to have a high index of suspicion for intestinal ischemia in methamphetamine users who present with acute abdominal pain.

## Highlights

• Methamphetamine is associated with non-occlusive intestinal ischemia.
• Splanchnic vasocontriction of mesenteric and colonic vessels is the most likely cause of methamphetamine-induced intestinal ischemia.
• Surgeons should have a high index of suspicion for intestinal ischemia in methamphetamine users who present with acute abdominal pain.

## 1 Introduction

Methamphetamine abuse is seen throughout Australia and has become a growing health concern. While acute psychosis is recognised as one of the most devastating health consequences of methamphetamine, its impact on physical health has been less appreciated. Recognizing methamphetamine-associated pathologies will allow clinicians to better approach conditions resulting from its use. There are well-documented cardiovascular and cerebrovascular pathologies of amphetamines, however, gastrointestinal pathology is less well understood. Methamphetamine can cause splanchnic vasoconstriction, so it follows that bowel ischemia may occur as a result. However, only a few case reports have confirmed this [[1], [2], [3], [4], [5]]. Herein we describe a rare case of intestinal ischemia due to methamphetamine use, managed at one of the metropolitan tertiary hospitals in Australia. The following case was written in line with the SCARE criteria [6].

## 2 Presentation of case

A 48-year-old man presented to an emergency department two hours after the intravenous administration and 3-day heavy use of crystal methamphetamine with crampy abdominal pain. He recalled the onset of pain was only half an hour after methamphetamine administration. His pain was mostly in the right lower abdomen and associated with emesis. He denied hematochezia, diarrhea, or fever. He admitted to having injected 4 points of amphetamine over two days, 2 points with each administration, prior to presentation. He had been using crystal methamphetamine intermittently for the past 20 years. His medical history is remarkable for hepatitis C for which he completed 12-week antiviral therapy, chronic back pain from previous motor vehicle accident, post-traumatic stress disorder, and previous appendectomy. He takes tramadol and paracetamol for pain if required. He smokes 20 cigarettes per week but does not drink alcohol.

On arrival at the emergency department he appeared alert and oriented with GCS of 15. His vitals were; temperature of 36.5 °C, blood pressure of 108/70 mmHg, pulse rate of 110 beats per minute, respiratory rate of 25 breaths per minute, and oxygen saturation of 99% on room air. The physical examination revealed mildly distended abdomen with diffuse tenderness, however, mostly in the right abdomen with guarding. The remainder of physical examination was remarkable, including a rectal examination.

Laboratory investigations revealed that the patient had a white cell count of 9.9 × 10^9^/L (ref range: 4.0–11.0 × 10^9^/L), haemoglobin of 162 g/L (ref range: 130–180 g/L), arterial pH of 7.24 (ref range: 7.35–7.45), base deficit of −8.3 mmol/L (ref range: −3.0 to 3.0 mmol/L), bicarbonate of 18 mmol/L (ref range: 22–32 mmol/L), lactate of 3.5 mmol/L (ref range: 0.2–2.0 mmol/L), CRP of 321 mg/L (ref range: <6 mg/L), creatine of 533 μmol/L (ref range: 60–110 μmol/L), eGFR of 10 (ref range: >59). Toxicology screening was not performed.

**Fig. 1:**
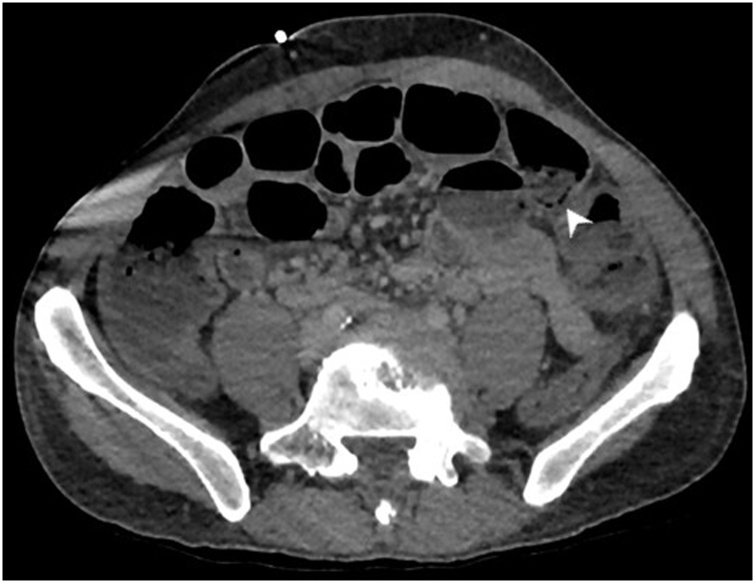
Contrast-enhanced CT scan of the abdomen demonstrated pneumatosis in multiple loops of ileum (white arrowhead).

**Fig. 2:**
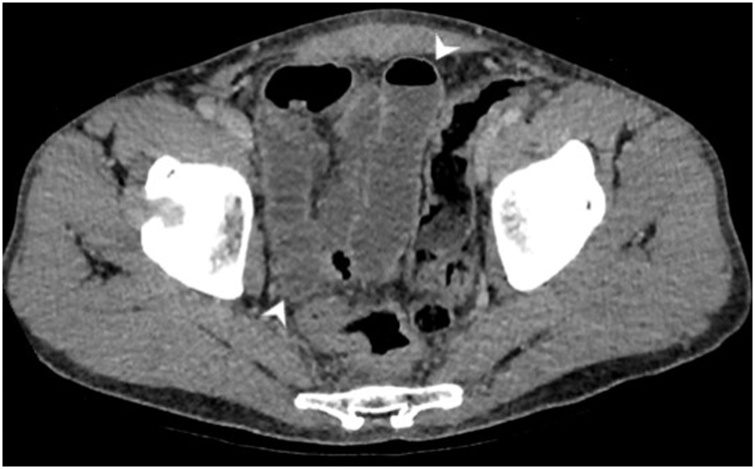
The ileum was also markedly dilated with reduced mucosal enhancement on portal venous phase imaging (white arrowheads).

**Fig. 3:**
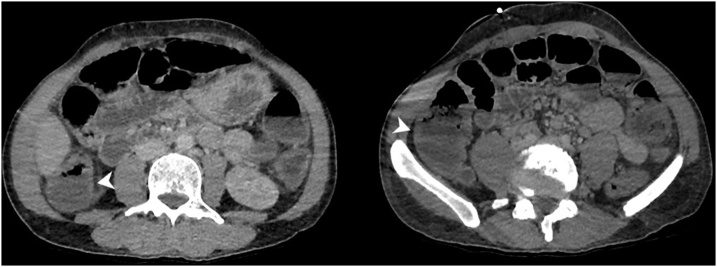
The ascending colon demonstrated segmental wall thickening without pneumatosis (white arrowheads).

Contrast-enhanced abdominal computed tomography (CT) showed dilated hypoenhancing ileum with pneumatosis intestinalis (Fig. 1, Fig. 2). It also revealed segmental thick-walled ascending colon (Fig. 3). The superior mesenteric artery (SMA) and stenotic inferior mesenteric artery (IMA) were patent without evidence of proximal thrombus.

With concern for bowel infarction he was taken urgently to the operating room for exploratory laparotomy and found to have caecal and ascending colonic gangrene and patchy necrosis of terminal ileum. However, he had a pulsatile superior mesenteric arterial flow. He underwent a right hemicolectomy and partial resection of terminal ileum as a damage control surgery. His bowel was left in discontinuity with a view of second-look operation in 24–48 h depending on his clinical condition. He returned to the intensive care unit for further resuscitation.

Pathological findings revealed segmental extensive mucosal ischemic necrosis, focal muscularis propria degeneration, marked wall oedema and a mild peritoneal reaction, but a lack of well-developed full thickness mural necrosis. Multiple small thrombi were present in mural and serosal venules, but extensive examination of the mesenteric vasculature failed to reveal either emboli or pre-existing vascular disease.

A second-look laparotomy was performed 24 h later: his remaining bowel appeared viable. Bowel continuity was restored by stapled side-to-side anastomosis.

Postoperatively he was continued on resuscitation at the intensive care unit; 4 days after admission his renal function normalized. He had uneventful recovery in the ward and was discharged day 13.

## 3 Discussion

The patient in this case presented with intestinal ischemia. A blood supply to small intestine and colon can be compromised either from occlusive or nonocclusive obstruction [7].

Occlusive obstruction is usually due to an arterial or venous embolism and/or thrombosis [8]. CT angiogram in this case did not reveal thrombi or emboli in mesenteric or colonic circulation. This implicates non-occlusive ischemia as a cause of intestinal ischemia.

Non-occlusive obstruction is most commonly caused by cardiac failure, arrhythmia, or hypovolemia [9]. The patient did not have symptoms or signs of cardiac failure or arrythmia. The patient was normotensive and clinically euvolemic. He was tachycardic but in sinus rhythm. Rarely nonocclusive mesenteric ischemia can be caused by use of vasoconstrictive agents, such as digoxin [10].

Methamphetamine is known to have sympathomimetic effect causing vasoconstriction in splanchnic circulation. Catecholamine release is increased with methamphetamine while its uptake at presynaptic axon terminals is reduced [11]. Excess catecholamines produce sympathomimetic vasoconstriction by acting on alpha-1 adrenergic in the arteriolar smooth muscle. Known cardiovascular and cerebrovascular complications include hypertensive crises, myocardial infarction, and cerebrovascular accidents [12,13]. The gastrointestinal complications, on the other hand, are not well documented. It is thought that gastrointestinal complication shares the same underlying mechanism. In theory severe reduction in mesenteric and/or colonic perfusion from vasoconstriction leads to bowel ischemia.

In this case, the most probable cause of bowel ischemia is methamphetamine-induced vasoconstriction. Temporal relationship and absence of thrombosis and vasculitis at the mesenteric and colonic vasculature support this presumptive aetiology. The patient’s gangrene was localized at ileum, caecum, and ascending colon. Those regions are supplied by superior mesenteric artery and its ileocolic and right colic branch. Intestinal ischemia can occur anywhere in the bowel. But the superior mesenteric arterial distribution is most commonly affected [14,15]. Colonic ischemia is, on the other hand, more transient and resistant to gangrene formation and tends to resolve without surgery or further complication [16].

Nonocclusive intestinal ischemia has a mortality rate of 70–90% [17]. Surgeons should have a high index of suspicion for bowel ischemia when methamphetamine users present with acute abdominal pain. Prompt diagnosis can possibly prevent complications and save bowel resection.

It should not be neglected, although we were unable to identify, that the patient could have had an undefined focus of infection that led to the poor perfusion of the intestine and other organ systems.

Methamphetamine is also known to cause intestinal dysmotility and ulcer formation with a few case reports confirming this [18,19]. However, there was no evidence to suggest these have occurred to the patient in this case.

## 4 Conclusion

We described the case of a 48-year-old man who presented with non-occlusive intestinal ischemia related to methamphetamine. This should alert surgeons to a high-index of suspicion for intestinal ischemia in methamphetamine users when they present with acute abdominal pain. A prompt diagnosis may reduce morbidity and mortality.

## Conflict of interest

I have nothing to declare in this category.

## Sources of funding

I have nothing to declare in this category.

## Ethical approval

Ethical approval was not required for this case.

## Consent

A written consent was obtained from the patient to write this case report using de-identified information and de-identified radiological images.

## Author’s contribution

Dr Kihoon Choi – collected and formally analyzed data; wrote the original draft.

Dr Andrew Imrie – interpreted radiological findings; provided relevant images.

Dr Rohan Lourie – interpreted histopathological findings.

Dr Trent Cross – reviewed and edited the original draft.

## Registration of research studies

I have nothing to declare in this category.

## Guarantor

Dr Kihoon Choi.

## Provenance and peer review

Not commissioned, externally peer-reviewed.
